# Supplementary material for: Effects of Traditional and Modern Post-Harvest Withering Processes on the Composition of the Vitis v. Corvina Grape and the Sensory Profile of Amarone Wines
Source: Molecules. 2021 Aug 27;26(17):5198. doi: 10.3390/molecules26175198 (PMC8434166; doi:10.3390/molecules26175198)

Effects of Traditional and Modern Post-Harvest Withering Processes on the Composition of the Vitis v. Corvina Grape and the Sensory Profile of Amarone Wines

Diego Tomasi [1], Andrea Lonardi [2], Davide Boscaro [1], Tiziana Nardi [1], Christine Mayr Marangon [3], Mirko De Rosso [1], Riccardo Flamini [1], Lorenzo Lovat [1] and Giovanni Mian [1,4*]

[1] Council for Agricultural Research and Economics – Research Centre for Viticulture and Oenology CREA-VE Viale XXVIII aprile 26, 31015 Conegliano, TV, Italy. diego.tomasi@crea.gov.it (D.T.); davide.boscaro@crea.gov.it (D.B.); tiziana.nardi@crea.gov.it (T.N.); mirko.derosso@crea.gov.it (M.D.R.); riccardo.flamini@crea.gov.it (R.F.); lorenzo.lovat@crea.gov.it (L.L.)

[2] Bertani Domains Società Agricola A R.L., Via Asiago 1, 37023 Grezzana (VR), andrea.lonardi@bertani.net

[3] Department of Agronomy, Food, Natural Resources, Animals and Environment (DAFNAE),University of Padova, Viale dell'Università 16, 35020 Padova, Italy, christine.marangon@unipd.it (C.M.M.)

[4] Department of AgriFood, Environmental and Animal Sciences, University of Udine, Via delle Scienze 206, 33100 Udine, Italy

* Correspondence: giovanni.mian@uniud.it

Supplementary Materials

Table S1. Stilbenes identified in Corvina grape before withering. Signal intensities are normalized to the internal standard.

stilbenes identified	signal intensity
trans-resveratrol	169027
piceatannol	490372
cis-piceid	34732
trans-piceid	35455
E-astringin	19778
Z-astringin	5072
pallidol	11191
resveratrol dimer	5555
Z-ε-viniferin	122415
Z-ω-viniferin	19796
E-ε-viniferin	582239
δ-viniferin	8816
caraphenol	24940
pallidol-glucoside	24020
α-viniferin	1848
Z- miyabenol C	4538
E-miyabenol C	26439
resveratrol tetramer 1	5076
resveratrol tetramer 2	16480

Table S2. Contents of glycoside precursors of volatile organic compounds (VOCs) identified in Corvina grapes harvested in 2016 and withered in controlled (C) and not-controlled (NC) environments (data expressed as µg 1-heptanol/kg d.g.).

	**(µg 1-heptanol/Kg d.g.)**													
	**T0**		**-10% WL**				**-20% WL**				**-30% WL**			
**2016**			**C**		**NC**		**C**		**NC**		**C**		**NC**	
	Mean	STD	Mean	STD	Mean	STD	Mean	STD	Mean	STD	Mean	STD	Mean	STD
aliphatic alcohols														
1-butanol	48	18	87	19	64	3	101	8	101	12	92	30	97	4
3-methyl-1-butanol	47	15	105	50	64	27	211	53	87	28	150	60	94	42
3-methy-3-buten-1-ol	25	4	33	3	30	6	36	4	38	3	33	11	36	1
1-pentanol	7	1	13	3	8	2	16	6	11	1	17	1	14	3
3-methyl-2-buten-1-ol	61	10	88	16	76	17	107	15	91	11	93	21	99	11
1-hexanol	69	18	77	19	56	4	110	18	74	17	113	71	119	55
3-hexen-1-ol (E)	1	0	1	0	1	0	3	1	1	1	2	1	2	1
3-hexen-1-ol (Z)	22	6	17	1	14	3	20	2	15	5	17	6	17	4
2-butoxyethanol	6	2	11	1	11	1	152	128	137	32	40	36	37	23
2-hexenol	11	2	12	2	11	2	16	9	15	5	18	8	18	2
C_6-aldehydes														
hexanal	13	3	23	2	29	11	27	11	25	11	13	5	20	3
2-hexenal	14	3	20	3	26	9	14	5	21	7	9	4	15	1
monoterpenes														
trans- furanlinalool oxide	2	0	3	1	2	1	3	1	3	1	3	1	3	2
cis- furanlinalool oxide	2	0	3	0	2	1	3	0	2	1	2	1	2	1
linalool	15	7	14	1	13	5	6	0	18	4	8	4	10	3
α-terpineol	3	0	5	1	3	1	5	1	4	1	4	1	4	3
trans-pyranlinalool oxide	8	2	11	2	8	5	12	2	11	4	11	4	11	7
nerol	16	1	24	4	18	4	21	6	22	2	19	6	20	1
geraniol	69	7	100	7	88	20	95	11	97	9	87	23	92	6
2-exo-2-hydroxycineol	9	3	11	2	11	5	13	3	13	4	14	7	13	4
diendiol I	10	3	10	2	11	5	10	3	12	4	11	6	9	3
trans-8-hydroxylinalool	39	17	73	17	50	13	68	21	61	4	77	7	59	20
hydroxygeraniol	19	2	37	6	26	8	44	3	36	5	37	10	41	5
cis-8-hydroxylinalool	104	31	132	24	90	48	128	33	143	20	134	32	125	45
geranic acid	56	6	116	11	91	12	107	11	120	9	106	25	116	9
7-hydroxy-α-terpineol	203	105	268	81	280	167	345	121	335	166	344	230	308	74
C_{13}-norisoprenoids														
3-hydroxy-β-damascenone	35	6	41	8	35	9	50	9	40	7	43	12	46	14
3-oxo-α-ionol	331	35	485	55	385	92	502	41	511	10	467	43	488	91
3,9-dihydroxy-megastigma-5-ene	28	5	35	4	26	6	38	3	35	1	37	6	36	8
3-hydroxy-7,8-dihydro-α-ionol	23	3	33	6	29	7	36	2	35	3	36	9	35	2
vomifoliol	368	81	510	115	439	92	524	85	556	39	543	90	538	107
benzenoids														
benzaldehyde	46	53	36	27	18	2	26	11	20	9	22	9	17	5
acetophenone	6	6	6	2	5	2	6	2	7	1	6	2	8	3
methyl salicilate	15	15	15	8	9	3	13	6	10	2	9	1	12	7
guaiacol	5	2	26	11	9	4	22	4	10	4	19	8	15	7
benzyl alcohol	590	173	799	207	1100	269	924	151	1345	590	964	213	1337	541
β-phenylethanol	276	26	391	96	404	85	478	64	447	63	418	135	436	39
eugenol	20	3	29	5	29	3	34	4	37	3	28	6	33	5
4-vinylguaiacol	375	76	808	186	800	107	993	68	900	42	841	201	999	161
syringol	58	12	275	86	83	41	228	70	87	33	176	75	127	56
4-vinylphenol	105	47	404	207	380	99	402	29	379	85	219	23	409	110
vanillin	30	6	54	15	48	5	66	8	51	6	56	16	55	2
methyl vanillate	42	15	54	13	51	21	67	16	59	13	59	23	53	8
acetovanillone	297	45	443	115	482	67	512	88	580	114	478	123	619	154
vanillic alcohol	267	48	620	335	625	42	760	213	724	155	601	111	815	243
homovanillic alcohol	196	46	416	134	394	51	437	85	509	59	467	131	539	11
4-hydroxy benzene ethanol	70	24	**260**	**107**	291	20	317	98	307	43	248	83	330	64
furanic compounds														
5-methylfurfural	27	8	43	7	42	3	48	6	57	1	43	15	54	1
2-furanmethanol	26	3	59	11	50	3	66	16	66	2	55	10	70	3
furaneol	**53**	9	102	18	92	5	114	22	123	1	99	22	124	4

Table S3. Contents of glycoside precursors of volatile organic compounds (VOCs) identified in Corvina grapes harvested in 2017 and withered in controlled (C) and not-controlled (NC) environments (data expressed as μg 1-heptanol/kg d.g.).

	(μg 1-heptanol/Kg d.g.)													
	T0		-10% WL				-20% WL				-30% WL			
2017			C		NC		C		NC		C		NC	
	Mean	STD	Mean	STD	Mean	STD	Mean	STD	Mean	STD	Mean	STD	Mean	STD
aliphatic alcohols														
1-butanol	74	24	76	17	68	9	76	9	94	49	112	41	174	58
3-methyl-1-butanol	79	19	117	111	76	16	99	30	118	54	161	109	225	39
3-methy-3-buten-1-ol	40	4	33	2	36	2	35	2	43	8	45	3	45	9
1-pentanol	11	5	5	0	7	2	8	1	10	4	8	1	16	7
3-methyl-2-buten-1-ol	108	14	101	11	104	12	108	19	136	30	112	17	87	77
1-hexanol	72	8	62	16	62	22	59	14	89	25	86	33	153	27
3-hexen-1-ol (E)	1	0	1	0	1	0	1	0	2	1	2	1	3	1
3-hexen-1-ol (Z)	33	4	16	3	23	10	13	5	21	4	14	9	23	2
2-butoxyethanol	62	45	123	69	78	96	51	32	30	15	95	79	76	71
2-hexenol	7	2	5	2	4	1	7	2	9	3	6	4	16	9
C_6-aldehydes														
hexanal	31	15	38	13	48	7	110	45	124	59	49	13	89	29
2-hexenal	28	7	18	6	22	2	11	4	14	1	16	4	16	2
monoterpenes														
trans- furanlinalool oxide	4	1	3	2	5	1	5	4	8	4	3	2	8	2
cis- furanlinalool oxide	3	0	3	1	5	2	6	4	8	4	3	2	8	3
linalool	49	12	34	11	40	4	36	19	39	6	19	7	39	37
α-terpineol	5	0	5	2	7	3	7	5	12	4	5	3	11	2
trans-pyranlinalool oxide	12	2	12	5	14	3	14	6	22	10	9	6	22	4
nerol	19	3	15	7	17	4	14	6	20	5	16	5	21	3
geraniol	79	14	66	28	76	19	61	26	82	19	80	23	87	21
2-exo-2-hydroxycineol	12	3	11	5	14	2	14	5	16	2	14	6	24	7
diendiol I	12	3	10	6	13	4	12	5	15	6	10	8	9	5
trans-8-hydroxylinalool	52	9	42	12	73	18	43	11	106	78	45	30	86	68
hydroxygeraniol	25	6	21	6	35	11	32	14	49	20	31	13	51	6
cis-8-hydroxylinalool	228	60	162	27	220	38	137	24	209	44	123	33	173	14
geranic acid	49	10	54	16	84	13	44	13	74	27	108	20	109	22
7-hydroxy-α-terpineol	188	46	185	120	225	30	214	92	251	45	232	117	249	31
C_{13}-norisoprenoids														
3-hydroxy-β-damascenone	51	7	55	8	64	5	66	13	84	22	47	10	109	46
3-oxo-α-ionol	541	63	466	178	737	49	733	150	878	214	562	100	1340	575
3,9-dihydroxy-megastigma-5-ene	31	3	35	5	36	4	31	5	41	17	25	9	40	6
3-hydroxy-7,8-dihydro-α-ionol	28	3	28	4	33	1	31	4	37	5	33	5	48	10
vomifoliol	422	101	377	52	592	79	623	202	928	308	497	99	1167	448
benzenoids														
benzaldehyde	39	17	30	7	20	2	50	19	32	15	34	16	43	25
acetophenone	6	1	5	2	5	4	8	3	7	6	6	3	15	14
methyl salicilate	4	2	6	1	3	1	8	3	6	1	6	0	24	18
guaiacol	125	24	111	104	139	32	243	259	576	324	222	192	799	266
benzyl alcohol	572	41	748	117	662	170	1263	259	1031	242	1484	360	1936	942
β-phenylethanol	370	41	412	99	456	49	368	54	453	113	457	90	593	60
eugenol	24	2	28	2	28	6	23	5	30	10	28	8	55	22
4-vinylguaiacol	578	99	493	120	583	85	467	94	746	196	736	159	1092	139
syringol	160	31	145	72	241	128	344	296	741	286	253	235	825	291
4-vinylphenol	265	126	135	103	228	54	281	129	579	219	688	312	513	192
vanillin	48	5	68	18	78	16	99	25	137	17	93	21	154	47
methyl vanillate	16	5	18	7	25	2	20	10	33	9	21	7	43	14
acetovanillone	268	24	240	192	377	68	321	14	467	132	340	61	744	262
vanillic alcohol	239	73	324	71	422	35	517	139	754	154	641	198	1079	430
homovanillic alcohol	349	65	381	67	358	114	390	42	518	136	518	61	624	93
4-hydroxy benzene ethanol	196	51	112	129	70	10	28	3	46	21	411	111	158	175
furanic compounds														
5-methylfurfural	9	5	7	1	6	1	7	1	7	2	7	1	9	3
2-furanmethanol	65	12	40	5	41	5	36	11	36	15	53	3	41	14
furaneol	29	6	29	5	20	10	25	4	26	4	45	8	32	9

Figure S1

On the left: “arelle” trays present in the non-controlled chamber, on the right trays in the controlled environment

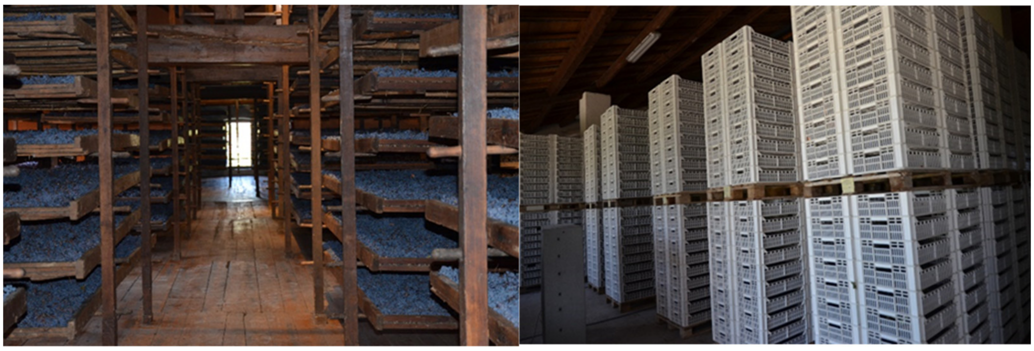

Figure S2

Sheet for wine sensory evaluation and related aroma proofile

SCHEDA DI ANALISI SENSORIALE O.I.V./U.I.Œ. **VINI TRANQUILLI** • FICHE D'ANALYSE SENSORIELLE O.I.V./U.I.Œ. **VINS TRANQUILLES**

N° campione/N° d'ordre de l'echantillon / Sample n°/N° de orden de la mustra ☐ — Commissione/Jury / Jury/Jurado ☐ — N° Commissario/N° de Dégustateur / Judge n°/ N° de Degustador ☐

		Eccellente<br>Excellent<br>Excellent<br>Excelente	Molto Buono<br>Très Bon<br>Very Good<br>Muy Bueno	Buono<br>Bon<br>Good<br>Bueno	Sufficiente<br>Satisfaisant<br>Fair<br>Regular	Insufficiente<br>Insuffisant<br>Unsufficient<br>Insuficiente
**COLORE<br>VUE<br>VISUAL<br>VISTA**	Limpidezza/Limpidité<br>Limpidity/Limpidez	☐ (5)	☐ (4)	☐ (3)	☐ (2)	☐ (1)
	Aspetto/Aspect (nuance)<br>Aspect/Aspecto	☐ (10)	☐ (8)	☐ (6)	☐ (4)	☐ (2)
**ODORE<br>ODORAT<br>NOSE<br>OLFATO**	Intensità/Intensité<br>Intensity/Intensidad	☐ (8)	☐ (7)	☐ (6)	☐ (4)	☐ (2)
	Franchezza/Franchise<br>Genuiness/Franqueza	☐ (6)	☐ (5)	☐ (4)	☐ (3)	☐ (2)
	Qualità/Qualité<br>Quality/Calidad	☐ (16)	☐ (14)	☐ (12)	☐ (10)	☐ (8)
**SAPORE<br>GOÛT<br>TASTE<br>GUSTO**	Intensità/Intensité<br>Intensity/Intensidad	☐ (8)	☐ (7)	☐ (6)	☐ (4)	☐ (2)
	Franchezza/Franchise<br>Genuiness/Franqueza	☐ (6)	☐ (5)	☐ (4)	☐ (3)	☐ (2)
	Qualità/Qualité<br>Quality/Calidad	☐ (22)	☐ (19)	☐ (16)	☐ (13)	☐ (10)
	Persistenza/Persistance<br>Persistence/Persistencia	☐ (8)	☐ (7)	☐ (6)	☐ (5)	☐ (4)
**ARMONIA<br>HARMONIE<br>HARMONY<br>ARMONIA**	**GIUDIZIO GLOBALE<br>JUGEMENT GLOBAL<br>OVERALL JUDGEMENT<br>APRECIACION GLOBAL**	☐ (11)	☐ (10)	☐ (9)	☐ (8)	☐ (7)

☐ Non degustabile/Non dégustable — Presidente ________ — Commissario ________

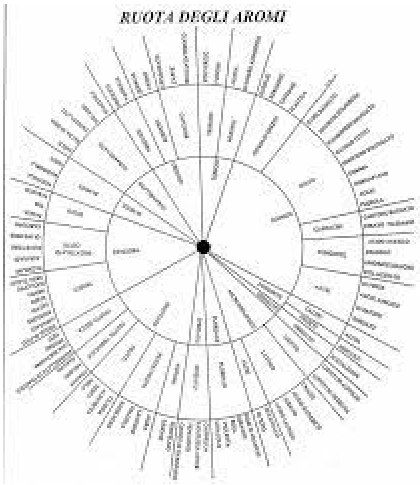

Supplement: Supplementary file 1 [file molecules-26-05198-s001.zip › molecules-1338770-supplementary.pdf]
